# Facilities for Fundamental Neutron Physics Research at the NIST Cold Neutron Research Facility

**DOI:** 10.6028/jres.098.010

**Published:** 1993

**Authors:** M. Arif, M. S. Dewey, G. L. Greene, W. M. Snow

**Affiliations:** National Institute of Standards and Technology, Gaithersburg, MD 20899

**Keywords:** beta decay, neutron decay, neutron interferometry, neutron lifetime, neutron wave, vibration isolation

## Abstract

The features of two fundamental neutron physics research stations at the NIST cold neutron research facility are described in some detail. A list of proposed initial experimental programs for these two stations is also given.

## 1. Introduction

In most of the experimental facilities at the CNRF, neutrons are used as probes to study structures and excitations in condensed matter. By contrast, the two research stations described in this article are devoted to the study of the neutron itself and its interactions. This area of research has come to be referred to as “fundamental” neutron physics.

The properties of cold neutron beams at guide halls are well-suited to fundamental neutron physics experiments. The slower average speed and consequent higher density of the neutrons in a cold beam (as compared to thermal and epithermal beams) result in improved accuracy in many measurements due to the longer interaction times with matter and external fields and the higher neutron decay activity per unit length. The relative ease with which cold neutron beams can be polarized and manipulated is very important for a number of experiments: in particular, it is crucial for experiments which search for effects due to parity and time reversal violation. Finally, the relatively quiet environment of a cold neutron guide hall (as compared to a reactor building) can lead to enhanced sensitivity in delicate experimental techniques such as neutron interferometry.

The fundamental neutron physics program at the NIST cold neutron research facility has two separate positions: A) A fixed neutron interferometer position, and B) an end guide position where appropriate instruments will be placed as needed to carry out various fundamental neutron physics experiments. We will first focus our attention on a general description of the neutron interferometry technique. A more detailed description of this technique can be found in references [1]–[7]

## 2. Neutron Interferometry

### 2.1 Background

**Fig. 1:**
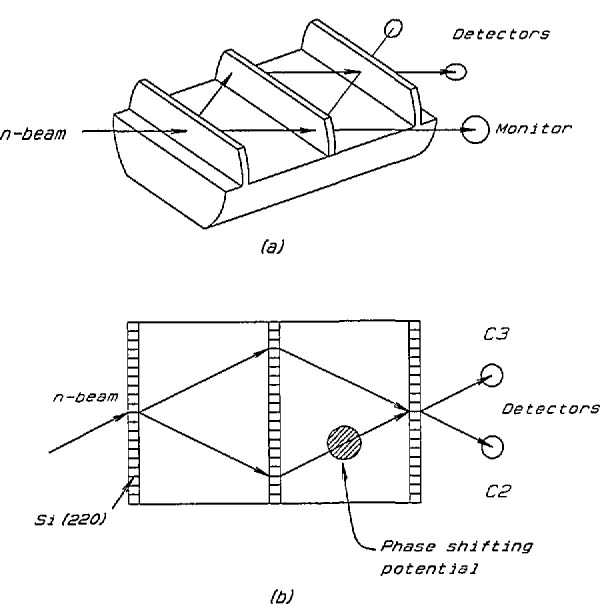
Schematic of ILL type neutron interferometer. Such a device was first tested by Rauch, Treimer, and Bonse in 1974.

A neutron interferometer is topologically analogous to the Mach-Zehnder interferometer of classical optics and operates on the basis of coherent splitting and recombination of the incident wave amplitude of a neutron wave through Bragg reflection in perfect crystals. Various arrangements have been realized to obtain interference effects between spatially separated coherent neutron beams. One of the most commonly used schemes is shown in Fig. 1a. The device consists of three very flat identical “ears” cut from a monolithic piece of silicon perfect single crystal leaving the three ears attached to a common base. The ears are usually cut perpendicular to strongly reflecting (220) lattice planes. Such a device is typically 50 to 100 mm in length and ears are typically 1 to 3 mm thick, 30 to 100 mm wide and 10 to 30 mm high.

In a neutron interferometry experiment the *phase* of a neutron wave becomes directly accessible. In this sense it is very different from other neutron experimental techniques where only the *intensity* of a neutron wave is accessible. In a simple manner the operating principle of such an interferometer can be understood from Fig. 1b. A nominally monoenergetic incident beam is coherently split by Bragg reflection in the first crystal ear (splitter). These two coherent beams are again split by the second ear (mirror) and finally two of the beams from the second ear overlap at the third ear (analyzer). If one of these interfering beams is phase shifted by an amount *β* with respect to the other beam due to interaction of some perturbing potential it can be shown that the expected intensities in the two detectors C2 and C3 are of the form,

I(C2)=A−Bcosβ (1)

I(C3)=B(1+cosβ), (2)

where *A* and *B* are constants depending on the properties of interferometer crystal and incident beam flux. The above equations predict that the sum of the intensities of the outgoing interfering beams is a constant. As a result the intensity is “swapped” back and forth between C2 and C3 and as evident from the above equations shows sinusoidal patterns. Relevant information about the interaction between the neutron wave function and the perturbing potential may be extracted from the phase, amplitude, and period of the intensity oscillation pattern.

The transit time of thermal neutrons through such a device is rather long (typically 30 to 100 μs) and there are perhaps 10^9^ quantum wavelengths in each path. To maintain the Bragg condition for a given wavelength of the neutron beam, the three ears must remain aligned within better than 10^−6^ rad. As a result neutron interferometry experiments are very easily adversely affected by environmental disturbances. Consequently, very stringent thermal, vibrational, acoustic, and geometric tolerances are required for successful operation of a neutron interferometer.

Research in neutron interferometry is primarily focused on three broad areas: the measurement of fundamental properties and interactions of neutrons, tests of fundamental propositions of quantum mechanics, and development of new measurement techniques. Since the first neutron interferometry experiment carried out by Rauch, Triemer, and Bonse in 1974, a large number of beautiful experiments have been performed to probe certain aspects of quantum mechanics which were previously either untested or indirectly inferred. The verification of the principle of equivalence in the quantum limit, the spinoral nature of fermions, and the Aharonov-Casher effect are some examples. A list of important neutron interferometry experiments in the last decade and a half is listed in Table 1. A list of proposed initial experiments for the CNRF interferometer is given in Table 2.

### 2.2 The MST Interferometer

#### 2.2.1 Interferometer Position

This position is situated in neutron guide number 7 (NG-7). The neutron interferometer experimental facility will consist of two major parts: (1) a double crystal monochromator assembly for extracting neutrons from the neutron guide, and (2) an environmentally controlled enclosure for experiments. These are discussed in the next few sections. In addition, a special interferometer mounting, neutron detection, and beam manipulation and analyzing setup will be built. A complementary x-ray setup will also be constructed for experiments and crystal diagnostics.

#### 2.2.2 Monochromator

**Fig. 2:**
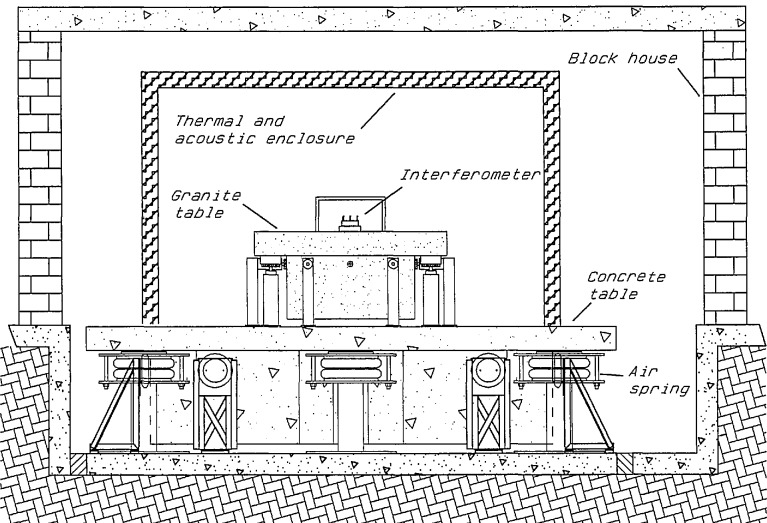
Schematic of the NIST interferometer setup.

**Fig. 3:**
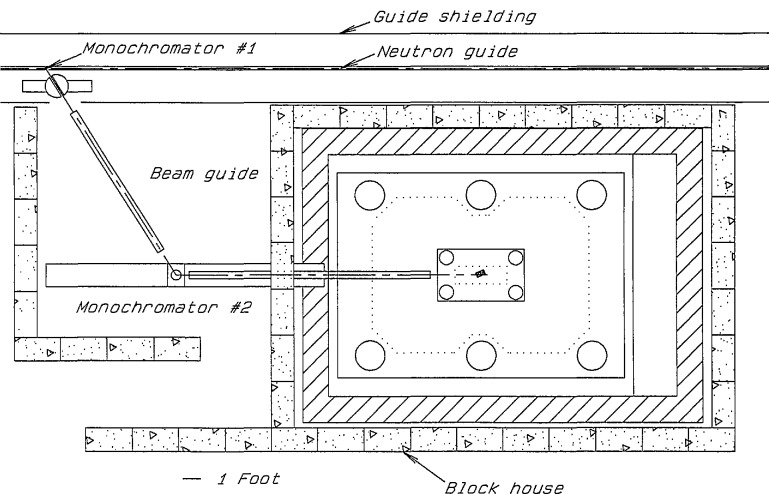
Diagram showing the double crystal monochromator layout for the NIST interferometer setup. The design of this monochromator system provides a unidirectional beam for the interferometer at all accessible incident neutron energies.

**Fig. 4:**
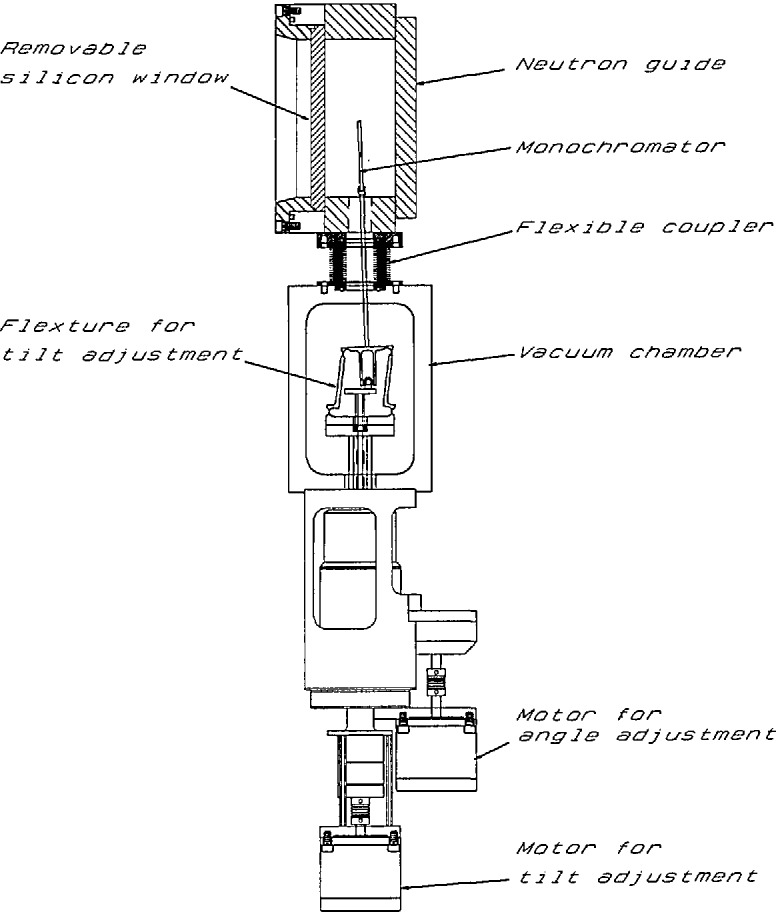
Diagram of the monochromator assembly which would allow rotation as well as tilting of a monochromator inside the guide.

System A variable-energy, monochromatic beam is provided by a double-crystal monochromator assembly. A schematic drawing of the overall layout of the interferometer station is shown in Fig. 2; a diagram of the double-crystal monochromator is shown in Fig. 3. This double-crystal arrangement allows the neutron beam to remain unidirectional at all attainable wavelengths. The range of 2*θ* angles is 40° to 90°, providing neutrons with energy range of 15.6 to 3.64 meV from pyrolytic graphite (002) and 47.7 to 11.2 meV from pressed silicon (220) monochromator crystals. The reflectivity of pyrolytic graphite (002) crystals in the range of wavelengths specified is 80% to 90%, while for pressed Si(220) it is expected to be 60% to 70%. Higher-order beam contaminants may be filtered-out with pyrolytic graphite or Be. The beam paths are necessarily rather long in this assembly. Therefore, to reduce attenuation due to air scattering and divergence, He-filled guide tubes will be employed. Each monochromator crystal is mounted on small high-precision rotary tables and positioned in angle, translation, and tilt by computer-driven stepping motors. This entire monochromator assembly is situated up-stream from the enclosure housing the interferometer vibration isolation platform discussed in the next section. The first monochromator crystal is mounted inside the neutron guide. This provides a significant reduction in background as the primary neutron beam does not pass through any windows or suffer air scattering between the primary guide sections. A novel monochromator support has been devised (Fig. 4) which allows manipulation of the horizontal tilt as well as the axial position of the crystal within the guide. The monochromator inside the guide has been installed recently. All its components have been tested and work well. A preliminary measurment shows a neutron flux of ∼ 1.0 × 10^6^ neutron-cm^−2^·s^−1^ at nominal 14.8 meV neutron energy. To the best of our knowledge this is the first and only monochromator operating inside a neutron guide.

#### 2.2.3 Environmental Control

Perfect crystal neutron interferometers are extraordinarily sensitive to environmental perturbations. Temperature gradients of millidegrees, displacements of fractions of an nm, velocities of (μm/s, rotational velocities of μrad/s, and vibrational amplitudes of nm are sufficient to seriously interfere with experiments. Obviously, these problems are more severe for the large, split-component (two or more pieces) interferometers. The major design goal for the interferometer station is to attain the best practical level of thermal, vibrational, acoustic, and seismic isolation. In the following we describe the environmental system designed to minimize environmental noise. The philosophy of the overall environmental system is based on the notion of “nested” isolation sub-systems. Experience has shown that it is difficult to obtain suitable isolation in a single “step”. An implementation of this philosophy is outlined in Fig. 2.

#### 2.2.4 Thermal Isolation

At least three levels of thermal control are envisioned. In each, a temperature control servo will be used to stabilize the ambient temperature: the first environment will consist of a large (∼ 6 × 8 × 3 m) concrete block house. The temperature of this large house will be thermostatically controlled by adjusting the flow of chilled water through radiators mounted on the interior house walls. Within this larger enclosure there is a smaller, acoustically isolated, quasi-hermetic room containing a monolithic granite surface plate with mass of ∼3000 kg. This enclosure has been designed and built. The temperature of this plate will be controlled by a heater fed back through an autotuning PID controller. By judicious selection of time constants, it will be possible to avoid coupled instabilities between these two nested servo loops. The third sub-environment will consist of a relatively small isothermal Aluminum Box whose temperature will also be controlled by a PID controlled Peltier heater/cooler.

#### 2.2.5 Vibrational and Seismic Isolation

If the interferometer were rigidly attached to the floor of the guide hall, it would be subjected to very significant accelerations (even micro *“g”* accelerations are significant). Therefore, major steps must be taken to decouple the interferometer from floor-born disturbances. Once again a system of nested isolation techniques has been employed. The first stage of isolation has already been provided for in the design of the guide hall building. The interferometer position is located on an independent foundation which is coupled to the rest of the building only through compliant joints (Fig. 2). Thus, the very significant amount of vibration associated with a reactor based experimental environment, which would normally be conducted along the path provided by continuous reinforced concrete, has been eliminated. It should be noted that no neutron interferometry experiments carried out thus far have had the benefit of such an independent foundation slab. In all other installations the interferometer position was located on a common foundation with reactor pumps, cranes, and heavy equipment. This feature of the proposed facility promises a very significant improvement over all previous installations. The second stage of vibration isolation is a pneumatically supported reinforced concrete slab of dimensions ∼5 × 4 × 1 m. This slab, with a mass of approximately ∼37000 kg rests on commercial pneumatic airspring supports. Such mass/spring systems can be conveniently modeled as damped harmonic oscillators. In this case the resonant frequency will be about 2 Hz. The response of such resonant systems to external driving forces at frequencies above the resonant frequency decreases logarithmically at about 6 dB/octave. Thus, such a system acts as a “low pass” filter for vibrational noise. High frequency vibrations will therefore be significantly reduced. In such a system it is important to insure that any internal resonances of the isolated mass have frequencies far higher than the characteristic mass/spring resonance of the isolator. If these criteria are not met, there may be significant coupling between the internal modes of the isolated mass and the “rigid body” excitations. The internal modes of the primary mass were estimated using dynamic finite element analysis. It has been determined that the lowest modes of the concrete slab lie in the range 80–100 Hz which is safely above the 2 Hz rigid body frequency. This second stage of vibration isolation has been installed recently. All its components and control system have been tested and this stage of vibration isolation has become operational. To further reduce vibration coupling to the interferometer, a second pneumatically supported granite table will be placed on top of the floating floor slab. This table has a mass of about 3000 kg and will be supported with a resonant frequency of about 0.5 Hz in the vertical and about 0.1 Hz in the horizontal directions. Because both the frequency and mass of this second suspended object are an order of magnitude less than the floating floor slab, coupled motions between the two coupled resonators will be small. In such a regime the overall isolation above the resonances will be multiplicative. Thus about 12 dB/octave roll-off in the high frequency regime can be expected. A practical problem associated with all pneumatic isolators arises from the intrinsic long term “softness” of such supports. A platform mounted on totally passive isolators (i.e., ones that are sealed) will exhibit long term positional drifts due to changes in ambient temperature, barometric pressure, or due to isolator leaks. Using a long time-constant electro-pneumatic servo mechanism (Ref. [7]), long-term positional stability of <10 μrad will be maintained.

#### 2.2.6 Acoustical Isolation

Unwanted instrumental vibrations can result not only from floor-born seismic noise, but also from airborne acoustic noise. To minimize coupling, both enclosures have been designed with careful attention to acoustic isolation. In addition they are lined with highly absorbing anechoic foam panels. Such panels are very effective in reducing reverberation times.

## 3. Fundamental Neutron Physics End-Guide Station

### 3.1 General

The end position on the NG-6 beam line at the CNRF is devoted to experiments in the field of fundamental neutron physics. The unique properties of the neutron which make it an ideal probe for a broad range of scattering experiments also make it an ideal system with which to address a number of important questions in nuclear physics, particle physics, and astrophysics. Typical experiments in the field include measurements of the static properties of the neutron, tests of fundamental symmetry principles, and investigations of neutron beta decay. The reader is referred to the proceedings of a recent conference in Ref. [5] to get a fuller view of the field.

In contrast to the neutron interferometry facility described in this paper, there is no permanently installed instrumentation or specific technique to be described. The properties of the beam must be matched to the requirements of each experiment individually. Aside from the beam shutter and a beam stop, the only other permanent equipment on the beamline is a filter cryostat to remove fast neutrons and gamma rays. A variety of collimators, polarizers, spin flippers, and transport tubes can be made available for individual experiments. The capture flux at the end of the 15 × 6 cm NG-6 guide has been measured to be about 4.0 × 10^+8^ neutrons/(cm^2^s), which is about a factor of nine lower than the 3 × 5 cm SN-7 guide at the ILL. This flux will increase significantly after the installation of the hydrogen cold source.

A measurement of the lifetime of the neutron is currently underway at NG-6. Testing of a prototype detector and polarizer system designed to search for time reversal invariance violation in neutron beta decay has been performed at NG-6. Other experiments in the near future may include searches for parity violation in neutron-nucleus scattering and studies of polarized neutron interactions with polarized nuclei.

#### 3.1.1 The Neutron Lifetime Measurement

We will describe the neutron lifetime experiment in some detail. This experiment developed from an earlier collaboration between NIST, the University of Sussex, and the Scottish Universities Research and Reactor Center, with related work on advanced methods of neutron flux measurements performed by NIST, the Central Bureau for Nuclear Measurements, the Scottish Universities Research and Reactor Center, Harvard University, and Los Alamos National Laboratory. A more detailed description than that given here can be found in Ref. [5].

A free neutron decays into a proton, an electron, and an electron antineutrino with a lifetime on the order of 900 s. There are a number of areas of physics for which a more accurate value for the neutron lifetime, τ_n_, is important. Within the context of the V-A theory of weak interactions a more accurate value for τ_n_, in combination with neutron decay asymmetry measurements, can provide accurate values for the weak vector and axial vector coupling constants *g*_A_ and *g*_V_. In an astrophysical context, the rate of energy generation in the sun is proportional to $g_{A}^{2}$, and a reduction in the accepted value of the neutron lifetime could contribute to an understanding of the solar neutrino problem. The neutron lifetime also has an important effect on the rate of helium production in the early universe (an important test of big bang nucleosynthesis) and on nucleosynthesis-derived limits on the number of light neutrino species, which constrain the number of generations in the present-day Standard Model of particle physics. The importance of these issues, coupled with the well-known inconsistencies in many earlier measurements, provide strong motivation for more accurate measurements of τ_n_.

**Fig. 5:**
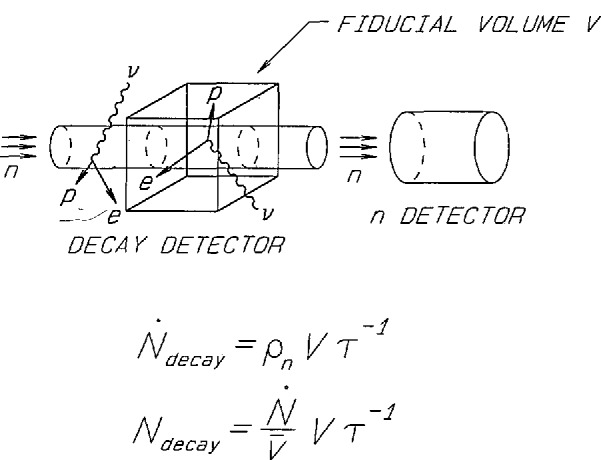
Illustration of the idea for the neutron lifetime measurement.

The strategy of our experiment is to measure the neutron decay rate *N*_decay_ and the mean number of neutrons *N*_n_ within a well-defined volume traversed by a cold neutron beam. The decay rate is related to these quantities by the differential form of the exponential decay law *N*_decay_=*N*_n_τ*_n_*^−1^. Figure 5 shows a schematic outline of the method. *N*_decay_ is measured in our experiment by trapping the protons produced in the decay with a Penning trap and counting the trapped protons. *N*_n_ is measured by requiring the neutrons leaving the decay volume to pass through an accurately calibrated neutron monitor.

The response of the neutron monitor is proportional to the beam flux weighted by the reciprocal of the velocity, in the absence of low energy resonances, the absorption cross section for neutrons is proportional to 1/*ν*. Since *N*_n_ is simply the beam flux integrated over the decay volume weighted by the reciprocal of the velocity (slower neutrons spend more time in the decay volume), the response of the beam monitor is proportional to *N*_n_. No detailed knowledge of the velocity distribution of the beam is necessary: however, it is necessary to measure the efficiency ∊_0_ of the neutron monitor at a single neutron velocity ν_0_. In terms of experimental quantities, the expression for the neutron lifetime becomes

$$\tau_{n}=\frac{N_{\alpha }L\in_{p}}{N_{p}\in_{0}V_{0}},$$ (3)

where *N_a_* is the count rate of the alpha particles produced by neutron absorption in the monitor, *L* is the length of the decay volume, and *N_p_* and ∊_p_ are, respectively, the count rate and the efficiency for the detection of the decay protons.

**Fig. 6:**
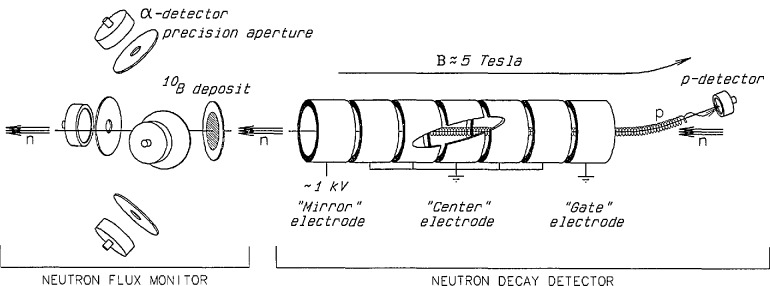
Schematic diagram of the neutron lifetime apparatus.

Figure 6 illustrates our realization of the strategy described above. The collimated neutron beam passes into the vacuum region and through the Penning trap, which is coaxial with the beam. The Penning trap consists of an axial magnetic field of approximately 5 T produced by a superconducting magnet along with an electrostatic potential well approximately 1 kV deep produced by a set of 16 isolated electrodes. The segmented electrode structure allows the decay volume to be varied electronically so that the ratio *N_p_/L* can be measured precisely: this procedure eliminates uncertainties in the size of the trapping volume due to unknown electric field end effects. Protons produced in the trap from neutron decay are confined radially in cyclotron orbits around the magnetic field lines. Since the maximum kinetic energy of the recoiling protons is 750.7 eV, the 1 kV electrode potentials confine the protons in the axial direction.

In order to count the protons, one end of the trap is opened by reducing the potential on an end electrode and producing a ramp in the potential with the middle electrodes. The protons then exit the trap and are guided along the magnetic field lines. A gentle 9° bend in the field lines guides the protons out of the neutron beam and toward a surface barrier detector held at a high negative potential (15–40 kV). The energy of the protons striking the surface barrier detector is high enough to produce a signal which is well separated from the electronic noise. The signal to noise ratio is further increased by gating the proton detector only while the trap is open.

The neutrons which pass through the trap strike the neutron monitor. The monitor consists of a thin silicon wafer with a thin deposit of ^10^B. Neutrons absorbed in the boron produce alpha particles which are detected by four surface barrier detectors.

The first experiment using this apparatus, performed at the ILL, measured a neutron lifetime of 893.6 s with a 1*σ* error of 5.3 s. This result is in excellent agreement with other recent direct measurements of the neutron lifetime using completely different techniques. In the next experiment, to be conducted at the NIST CNRF, we hope to reduce the 1*σ*-error to 1.5 s. Table 3 shows the error budget for the ILL experiment (phase 1) and the anticipated error budget for the NIST CNRF experiment (phase 2). Most of the improvement in the anticipated error comes from better counting statistics and improvements in the calibration of the neutron monitor detector efficiency.

In the ILL experiment, the neutron monitor efficiency was calculated using measurements of the detector solid angle, the cross section for neutron absorption in ^10^B, and the mass per unit area of the boron deposit. For the NIST measurement, we intend to determine e*ϵ*_0_ by calibrating the monitor in a monochromatic beam with accurately known velocity *ν*_0_ against two totally absorbing (“black”) neutron detectors. One of the black detectors is a cryogenically cooled neutron calorimeter which can determine the neutron flux by measuring the heat produced by neutron capture in a totally absorbing ^6^LiPb target. The other instrument is a totally absorbing capture gamma detector, whose efficiency is determined directly by alpha-gamma coincidence counting and indirectly by comparison with standard alpha sources. Each of the methods has a potential accuracy at the 0.1% level. If the uncertainty in *ϵ*_0_ can be reduced by calibration with the black detectors, the neutron lifetime measurement at the NIST CNRF may turn out to be the most accurate measurement of the neutron lifetime.

#### 3.1.2 Time Reversal Symmetry Violation in Neutron Decay

A prototype detector and polarizer system designed to search for time reversal violation in neutron decay was tested in spring 1992. The prototype has been constructed as a collaborative effort by Los Alamos National Laboratory, Harvard University, Argonne National Laboratory, the University of Michigan, the University of California, and NIST. The goal of the measurement is to improve by an order of magnitude the sensitivity to *T* violation in neutron decay through a possible term in the decay probability which is proportional, in the nonrelativistic limit, to the triple correlation *σ*_n_ ·[*P_e_* × *P_ν_*]. This possible time reversal violating term in neutron decay is known as the *D* coefficient.

The only experimental evidence for time reversal violation found to date lies in the dynamics of the neutral K-meson system. Although the standard model of particle physics with three generations of quarks and leptons can accommodate the possibility of time reversal violation in the K-meson system, it is still not known whether or not theory and experiment are in quantitative agreement. The observation of time reversal violation in any other system would be a result of fundamental importance. The observation of a non-zero value for *D* in the 10^−3^−10^−4^ range, which should be within the sensitivity of this experiment, would almost certainly indicate the presence of physics beyond the standard model.

The actual *T* violation measurement concerns the correlation between the neutron spin (*σ*_n_) and the decay proton and electron momenta (*p*_P_,*p*_e_). The triple correlation involving the neutrino momentum *p_v_* can be inferred from this measurement. The experimental apparatus consists of two major components: a neutron polarizer to fix *σ*_n_ and a coincidence detector capable of simultaneously determining *p*_p_ and *p*_e_.

The experiment will employ a novel method of neutron polarization based on transmission of neutrons through spin polarized ^3^He. The neutron cross section for ^3^He is very spin dependent: for thermal neutrons the cross sections are 5327 b for antiparallel spins and only a few b for parallel spins. The ^3^He will be polarized by spin exchange with optically pumped rubidium in a vapor cell. Such a polarizer is ideally suited for polarized neutron decay experiments. It will produce much less background due to absorption in the “wrong” spin state than more conventional polarizers and therefore a much lower rate of false coincidences in the decay detector. In addition, such a ^3^He polarizer will not significantly increase the divergence of the neutron beam, in contrast to conventional magnetic mirror devices.

The coincidence detector will consist of alternating electron and proton detector panels arranged in an octagonal array surrounding the neutron beam. The electron detectors will be plastic scintillators. The proton detector panels will consist of an array of silicon detectors biased at 30−40 kV with electrostatic lenses for focusing. The proton detector will provide directional information only: from extensive Monte Carlo simulations it has been determined that the absence of proton tracking only minimally reduces the sensitivity of the experiment. This detector arrangement was successfully tested in spring 1992.

## 4. Conclusions

The NIST CNRF will provide new opportunity to researchers in traditional areas of materials, structural, and dynamic applications. However, the spectrum of research will not be limited only to these areas, but will also expand to experimental inquiry in which low energy neutrons will be used for the investigation of fundamental interactions. The proper development of these fundamental physics research stations will be vitally important for the growth and successful continuation of the programs in this field. We envision that when fully developed, the experimental facilities provided by these two stations will be comparable or better than similar facilities elsewhere. It is hoped that the research facilities at these two positions will enhance and stimulate further research work in topics of fundamental interest such as parity and time reversal symmetry violation, baryon nonconservation, weak interactions, fundamental constants, charge conservation, and various other quantum mechanical phenomena. Results of these works will have important implications in particle and nuclear physics, astrophysics and cosmology, and fundamental quantum theory.

The end position has recently become operational and as mentioned earlier a measurement of the neutron life time is already underway. The major design works of the neutron interferometer station have been completed or are nearing completion. It is expected that a preliminary neutron interferometer setup will become operational in 1993.

## Figures and Tables

**Table 1:** Neutron Interferometry (1974–1990)

1.	First test of Si-crystal interferometer.
	Vienna (1974).
2.	Sign change of fermion wave function during 2*π* precession.
	ILL,^a^ MURR^b^ (1975, 1976).
3.	Gravitationally induced quantum interference.
	Ann Arbor, MURR (1975, 1980, 1985, 1986).
4.	Neutron Sagnac effect.
	Earth’s rotation. MURR (1979).
	Turntable MIT^c^ (1984).
5.	Neutron Fizeau effect.
	Moving boundaries. ILL (1981, 1985).
	Stationary boundaries, MURR (1985, 1988).
6.	Search for non-linear terms in the Schrödinger equation.
	LL interferometer. MIT (1981).
	Long wavelength fresnel diffraction. ILL (1981).
7.	Search for Aharonov-Bohm effect for neutrons.
	MIT (1981).
8.	Measurement of neutron longitudinal coherence length.
	MURR (1983).
9.	Coherent superposition of spin states.
	(“Wigner phenomena”) ILL (1983).
10.	Search for quaternions in quantum mechanics.
	MURR (1984).
11.	Quantum interference in accelerated frames.
	ILL (1983).
12.	Search for new gauge fields.
	MIT (1983).
13.	Precision measurement of scattering lengths.
	^149^Sm, ^235^U, ^3^He, ^3^H (four body nuclear reaction).
	ILL, MURR (1975–1985).
14.	Observation of the Aharonov-Casher effect.
	MURR (1989).
15.	Stochastic vs. deterministic attenuation of neutrons.
	ILL (1987).
16.	Neutron spin-pendellosung resonance.
	MIT (1988).

b MURR —Unversity of Missouri Research Reactor.

c MIT —Massachusetts Institute of Technology.

a ILL —Institut Laue-Langevin.

**Table 2:** Initial scientific programs

End position	Interferometer position
1. Determination of neutron lifetime.	1. Michelson-Morley experiment.
2. Time reversal in neutron-decay.	2. Wheeler delayed choice experiment.
3. Parity violation in neutron-nuclear scattering.	3. Precision Aharonov-Casher experiment.
4. Measurement of fundamental constants of neutron.	4. Search for Mahshoon effect.
5. Determination of neutron-decay coefficient.	5. Large-scale interferometer development: Cavendish experiment.
6. Studies of polarized neutron interaction with polarized nuclei.	

**Table 3:** Error estimation

Source of Error	Phase 1 (s)	Phase 2 (s)
Statistics of decay detection	3.8	<10
^10^B-foil mean mass per unit area (IDMS)	2.8	NA
^10^B cross section	1.4	NA
^10^B-foil central shape correction	0.4	NA
^10^B-foil central shape x beam profile	0.4	<0.1
^10^B-foil absorption	0.7	0.1
Si-backing absorption	0.2	0.1
Si-backing incoherent scattering	0.5	0.2
α-detector solid angle *Ω*	1.0	NA
α-detector *σΩ* due to beam profile	0.3	0.1
p backscattering from Au	1.0	0.2
Near normal forward scattering of protons	0.1	0.1
Si-p backscattering	05	0.2
Trap length 20°C	0.5	0.2
Timing of mirror electrode	0.5	0.3
Trapable background	<0.1	<0.1
Trap thermal contraction	<0.1	<0.1
Si-backing Bragg Scattering	<0.1	<0.1
*Ω* (B) modification of trajectory	<0.1	<0.1
Neutron trajectory in trap	<0.1	<0.1
Trap alignment	<0.1	<0.1
Diffusion loss of protons	<0.1	<0.1
Neutron beam centroid misalignment	<0.1	<0.1
Trap end effect and beam divergence	<0.1	<0.1
Calibration of neutron monitor	NA^a^	≈ 1.0

Total error	5.3	≈1.5

a Not Applicable.
